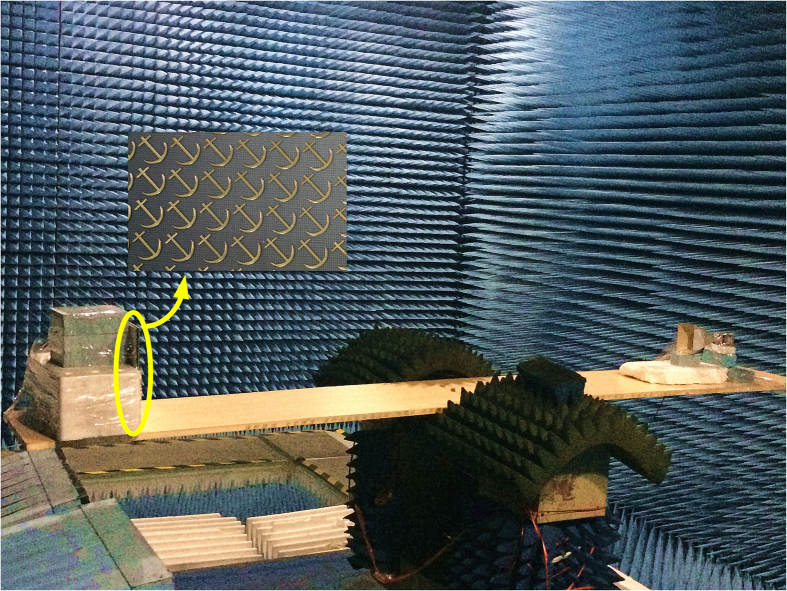# Ultra Wideband Polarization-Selective Conversions of Electromagnetic Waves by Metasurface under Large-Range Incident Angles

**DOI:** 10.1038/srep12476

**Published:** 2015-07-23

**Authors:** Jia Yuan Yin, Xiang Wan, Qian Zhang, Tie Jun Cui

**Affiliations:** 1 State Key Laboratory of Millimeter Waves, Southeast University, Nanjing 210096, China; 2 Synergetic Innovation Center of Wireless Communication Technology, Southeast University, Nanjing 210096, China

## Abstract

We propose an ultra-wideband polarization-conversion metasurface with polarization selective and incident-angle insensitive characteristics using anchor-shaped units through multiple resonances. The broadband characteristic is optimized by the genetic optimization algorithm, from which the anchor-shaped unit cell generates five resonances, resulting in expansion of the operating frequency range. Owing to the structural feature of the proposed metasurface, only *x*- and *y*-polarized incident waves can reach high-efficiency polarization conversions, realizing the polarization-selective property. The proposed metasurface is also insensitive to the angle of incident waves, which indicates a promising future in modern communication systems. We fabricate and measure the proposed metasurface, and both the simulated and measured results show ultra-wide bandwidth for the *x-* and *y-*polarized incident waves.

Metamaterials are artificial materials with unusual electromagnetic responses that are not possessed by natural materials^1^,^2^,^3^,^4^,^5^,^6^. In the past decades, researchers have engaged in planar metamaterials composed of subwavelength periodic resonant or non-resonant unit cells^7^,^8^,^9^, which are also called as metasurfaces. Some important applications of metasurfaces are anomalous refraction or reflection^10^,^11^,^12^, surface-wave conversion^13^,^14^, and polarization transformation^15^,^16^,^17^. Ref. 18 presented a general view of various kinds of metasurfaces and reviewed recent developments in this area. Among all the functional metasurfaces, the free control and manipulation of the polarization is of great significance. Khanikaev *et al.* have demonstrated polarization conversion and electromagnetically induced transparency with metasurfaces by using the interference of two polarization-selective plasmon resonances^19^. Pors *et al.* have studied metasurface-based beam splitters operating in reflection mode^20^. They used a gap surface plasmon resonance to control the phase of the reflected wave. Metasurfaces with different phase patterns for TM and TE polarizations can be designed by adjusting the resonance position with proper geometric parameters. This will result in distinct behaviors of differently-polarized components. Moreover, Pfeiffer *et al.* have proposed a bianisotropic metasurface which allows for a strong polarization control of light^21^. The proposed structure owns an improvement in comparison to previously results. There are also plasmonic metasurfaces employed to control the circular polarization of thermal radiation^22^,^23^. Left- or right-circularly polarized thermal radiation can be emitted from the surface because of the spin-orbit interaction of the thermal radiation with the metasurface. Owing to the unusual properties of metasurfaces, some problems of conventional polarization-control materials, such as the thickness of the structure and bandwidth, can be smoothly solved.

Miniaturized polarization controllers have spurred long-held interest since the birefringence effect of anisotropic metasurfaces^24^,^25^,^26^ and the optical activity of chiral metasurfaces^27^,^28^,^29^ had been further investigated. However, the bandwidth expansion is still a main concern that needs promotion. There are mainly two methods to broaden the bandwidth, one of which is stacking metallo-dielectric multilayers. An ultrathin multilayer-stacked system has been proposed by containing twisted complementary split-ring resonators (CSRR) for highly efficient broadband polarization transformation^30^. The operating frequency range is broadened to about 9.8–12.5 GHz, which is at the expense of bulkier structure. The other method is using multiple resonances^17^, in which anisotropic high-impedance surfaces are used to achieve perfect polarization rotation and the operating frequency band is broadened by high-order plasmon resonances. The structure can rotate the polarization plane in a broadband of 2.0–3.5 GHz, in which different resonances at three frequencies are generated. Recently, an ultra-wideband polarization-conversion metasurface has been proposed through four electric and magnetic resonances^31^, which can achieve a 1/4 3 dB bandwidth for both normally incident *x-* and *y-* polarized waves. However, the polarization conversion ratio is only higher than 50%^31^.

In this article, we propose an ultra-wideband polarization-conversion metasurface with the polarization selective and incident-angle insensitivity characteristics using an anchor-shaped unit through multiple resonances. The broadband feature is optimized using the genetic optimization algorithm. The anchor-shaped unit cell can be regarded as a composite of transformed V-shaped resonator and cut-wire resonators, both of which have been demonstrated to rotate the linearly polarized electromagnetic wave to its orthogonal direction. The electric and magnetic responses of the anchor-shaped unit cell generate five resonances, resulting in expansion of the operating frequency range. The proposed ultra-wideband polarization-conversion metasurface is fabricated in an area of 210 *mm* × 210 *mm*. Both simulated and experimental results indicate that the proposed metasurface owns a wide bandwidth for both normally incident *x-* and *y-* polarized waves. The average polarization-conversion ratio is higher than 80% within the 3dB bandwidth, and the efficiency is nearly 100% at five resonance frequencies. Meanwhile, when the incident waves are not *x*- or *y*-polarized, the conversion ratio remains low. This phenomenon indicates that the proposed metasurface has a polarization selective feature that only the *x-* and *y-*polarized waves can be converted, reducing interference among the cross-polarized waves in the communication system. When the incident waves are not normally incident, the proposed metasurface still performs well in the working band, providing convenience in practical applications.

## Results

**Figure 1:**
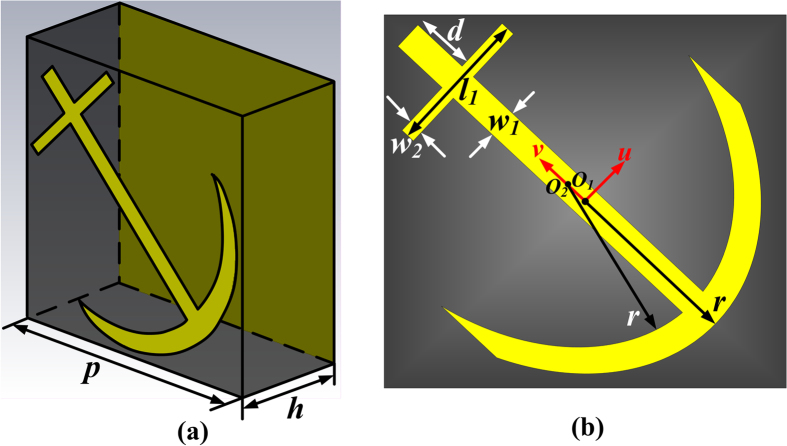
Schematic of the anchor-shaped unit cell. (**a**) The yellow parts are metal (modeled as copper) and the grey part is the 3mm-thick substrate. (**b**) Specific illustration of the anchor-shaped unit cell, in which *O*_*1*_and *O*_*2*_bear a distance of 0.6 *mm* along the *v*-axis. The angle of the transformed V-shaped resonator is *θ*, which is not labeled in the figure. (This figure was drawn by Jia Yuan Yin)

The proposed ultra-wideband polarization-conversion metasurface is composed of anchor-shaped array and a copper ground sheet. The anchor-shaped array is fabricated on a 3 mm-thick substrate with a relative permittivity of 2.65 and loss tangent of 0.001. Figure 1 gives the schematic of the anchor-shaped unit cell with the periodicity *p* = 7*mm*. The thickness of metallic layer *t* is 0.018 *mm*. The specific values of other parameters will be illustrated in the following discussions combined with the introduction of genetic optimized algorithm.

To understand the principle of the polarization conversion, the anchor-shaped unit cell can be regarded as an anisotropic homogeneous material layer with the thickness *h + t*, the relative permittivity ε, and a dispersive relative permeability tensor 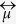 . The anchor-shaped unit cell has a symmetrical axis, labeled as the *v*-axis, along 45° direction with respect to the *y*-axis, as shown in Fig. 1. Here, 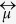 can be expressed by diagonal elements (*μ*_*uu*_, *μ*_*vv*_, *μ*_*zz*_) employing *u*, *v* and *z* as the orthogonal coordinate system.

**Figure 2:**
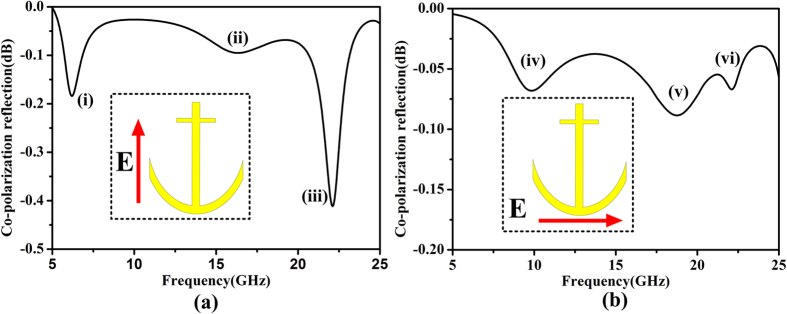
Current distributions at corresponding resonances. (**a**) *V*-polarized case. (**b**) *U*-polarized case.

The anchor-shaped resonator can be seen as the combination of a transformed V-shaped resonator and cut-wire resonators, as shown in Fig. 1. According to the analysis in Ref. 10, the V-shaped resonator supports “symmetric” and “anti-symmetric” modes, excited by electric-field components along *v*- and *u*-axes, respectively. The electric-field components along the *v*-axis can also excite multi-order dipolar resonances on cut-wire resonators. Thus the combined structure is supposed to have multiple resonances. To verify this assumption, we employ the commercial software, CST Microwave Studio, to simulate the resonant eigen-modes of the anchor-shaped resonator. It is obviously that the resonant eigen-modes can be excited by the electric-field components along *u*- and *v*-axes. Figure 2 presents the simulated results, indicating that there are six dips. The three eigen-modes (i), (ii) and (iii) are excited by *v*-polarized EM waves, as shown in Fig. 2(a), while the three eigen-modes (iv), (v) and (vi) are excited by *u*-polarized EM waves, as shown in Fig. 2(b). Among all resonances, (iii) and (vi) arise at the same frequency practically. Thus when the *y*-polarized waves are incident, five resonances can be excited since the *y*-polarized waves have both *u*- and *v*-components simultaneously. Without the backing metal ground sheet, these resonances still exist but are quite weak.

**Figure 3:**
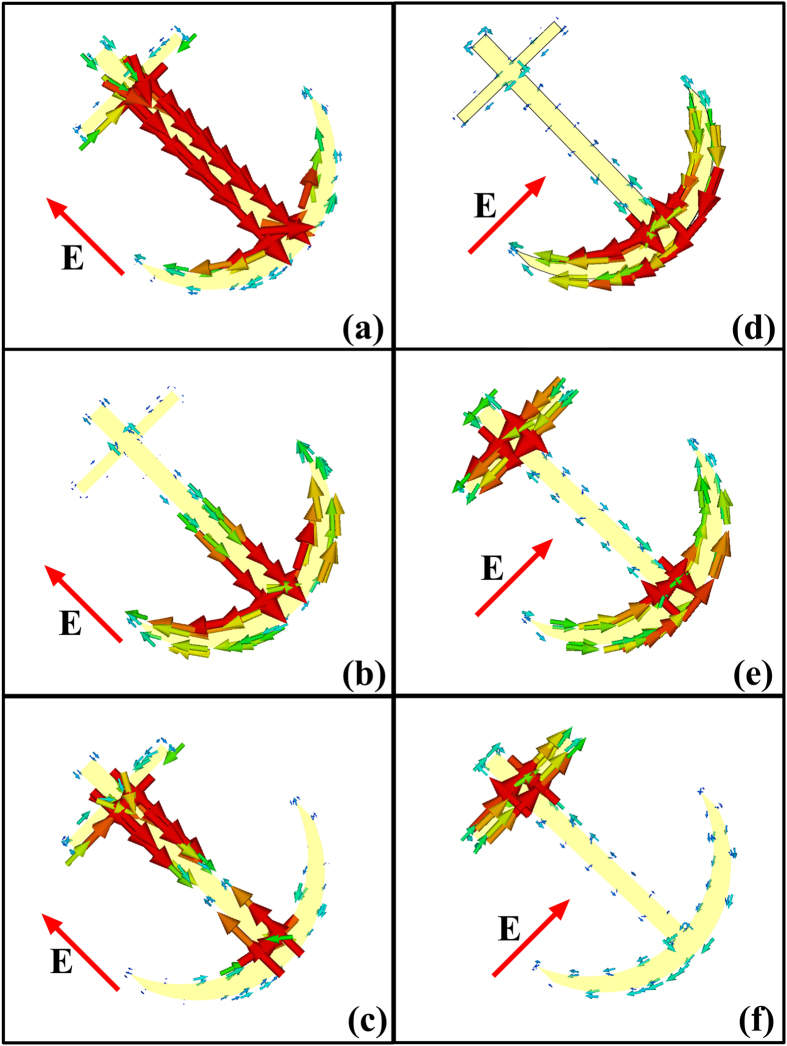
Resonant eigen-modes of the anchor-shaped unit cell under the normal incidence. (**a**) 9 GHz. (**b**) 19.6 GHz. (**c**) 22.3 GHz. (**d**) 6.6 GHz. (**e**) 16 GHz. (**f**) 22.3 GHz.

To have an intuitive understanding of the resonances, Fig. 3 gives the current distributions at the corresponding resonances, in which (a)–(c) correspond to the consequences with *v*-polarized incident waves, while (d)–(f) correspond to the *u*-polarized incident waves. As demonstrated in Fig. 3, the “symmetric” and “anti-symmetric” modes are excited on the transformed V-shaped resonators, and the multi-order modes are excited on the cut-wire resonator.

According to Faraday’s law, surface currents of opposite directions are induced in response to the time-varying magnetic field sandwiched between the structure and metal ground, and hence the magnetic responses are generated. At the resonant frequencies, μ becomes very large, resulting in a very large surface impedance 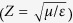 and in-phase reflection^32^,^33^. When the polarization direction of the incident EM waves is along the *u*-axis, odd-order resonances are generated; when the polarization direction of the incident waves is along the *v*-axis, however, even-order resonances are produced. We remark that *μ*_*uu*_ and *μ*_*vv*_ are different since the structure presents anisotropic property. Hence, at the resonant frequency, the structure acts as a high-impedance surface to present the in-phase reflection in one direction, while produce the out-phase reflection in the other direction.

**Figure 4:**
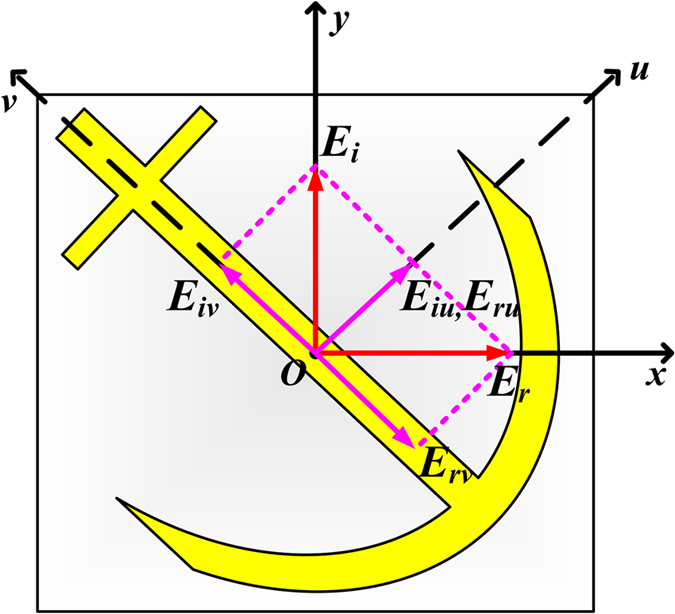
Decomposition of electric vectors of incident and reflected EM waves on *z* = 0 plane .

Take the *y*-polarized incident EM waves as an example. As shown in Fig. 4, the electric field vector at *z* = 0 plane can be decomposed as 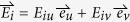, where 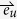 and 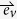 are the unit vectors in the *u*- and *v*-axes, respectively. At the resonance frequency, the components of the reflected EM waves *E*_*ru*_ and *E*_*rv*_ take a 180° phase difference, leading to the *x*-polarized reflected EM waves 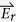.

**Figure 5:**
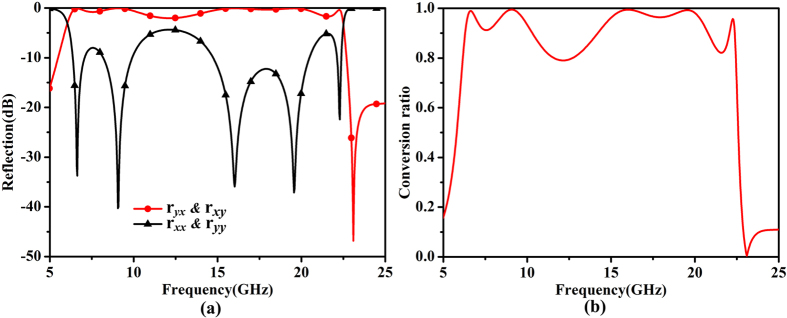
Simulated results of the proposed anchor-shaped unit cell. (**a**) The co- and cross-polarized reflection coefficients. (**b**) The polarization conversion ratio.

Figure 5 shows the simulated cross- and co-polarized reflections and polarization-conversion ratios with the optimal parameters. It is obviously that the cross-polarization reflection band is very wide, as illustrated in Fig. 5(a). The 3 dB bandwidth is ranging from 6 to 23 GHz for both *x*- and *y*-polarized incident EM waves. The wide band results from the five resonances as discussed above. The resonant frequencies are 6.6 GHz, 9 GHz, 16 GHz, 19.6 GHz, 22.3 GHz, at which the polarization conversion efficiency is nearly 100%. Within the 3dB bandwidth, the polarization conversion efficiency is higher than 80%.

**Figure 6:**
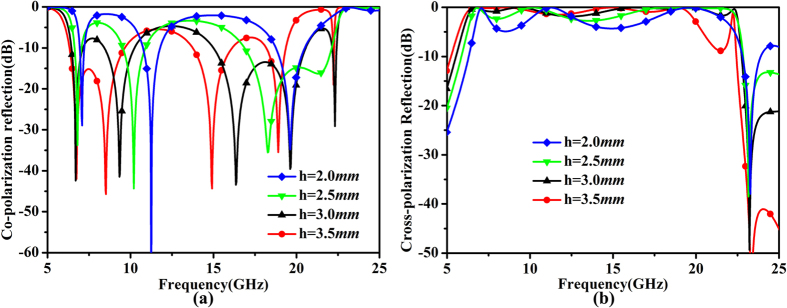
The simulated results for different heights of the substrate. (**a**) Co-polarization reflection coefficients. (**b**) Cross-polarization reflection coefficients.

The height of the substrate is another factor affecting the bandwidth and performance of the proposed metasurface. From the simulated results shown in Fig. 6, it is clearly that the bandwidth is broadened as the substrate thickness increases. However, when the substrate becomes thicker than a certain value, the performance of the metasurface becomes worse. Thus both bandwidth and performance should be taken into consideration when designing the polarization-conversion metasurface.

**Figure 7:**
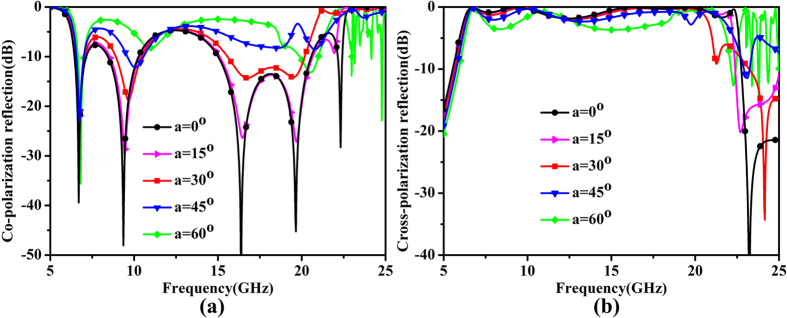
The simulated results for different incident angles. (**a**) Co-polarization reflection coefficients. (**b**) Cross-polarization reflection coefficients.

To find out the relationship between the incident angle and the conversion performance, we change the incident angle in the simulation procedure. Figure 7 gives the simulated results for different incident angles. It can be seen that the performance becomes worse as the incident angle increases but still remains well generally in the working frequency band. The incident angles have little effects on the bandwidth and performance. Such insensitivity to the incident angle provides convenience in practical applications. That is to say, when we use the proposed metasurface to convert the polarization of incident waves, it is unnecessary to use the normal incidence rigorously, which is often hard to achieve in reality.

**Figure 8:**
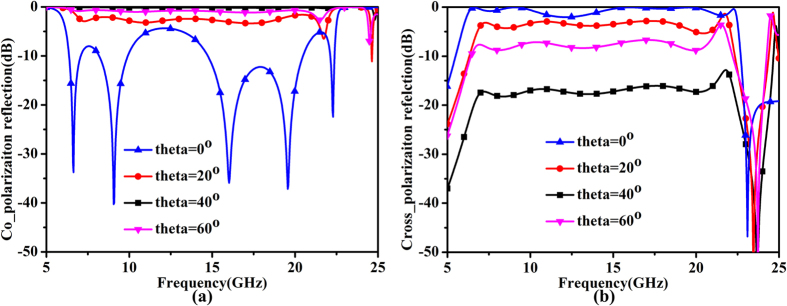
The simulated results for differently polarized incident waves. (**a**) Co-polarization reflection coefficients. (**b**) Cross-polarization reflection coefficients.

Another advantage of the proposed metasurface is its polarization-selective characteristic: only the *x*- or *y*-polarized incident wave can be converted to its orthogonal direction. When the incident wave holds an angle to the *x*- or *y*-axis, the polarization conversion is very weak. Figure 8 is the simulation proof of this phenomenon, in which *theta* is the angle of the incident wave with respect to the *y*-axis. When *theta* is further away from the *y*-axis, there is no apparent resonance in the operating frequency band. Hence the proposed metasurface has little effect on the incident waves. This characteristic can reduce the interference among the cross-polarized waves in the modern communication system.

**Figure 9:**
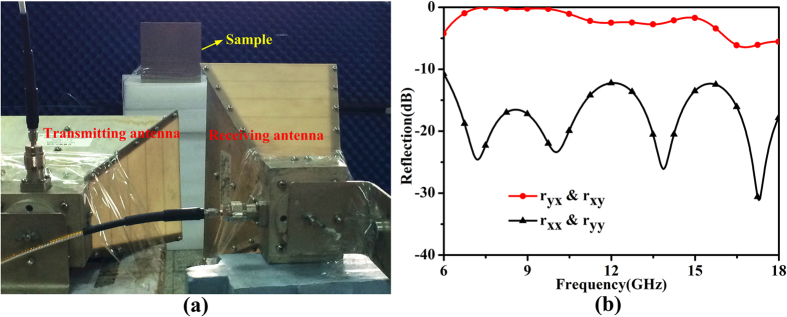
The measurement setup and measured results. (**a**) The experimental platform constructed for measurement. (**b**) The measured co- and cross-polarized reflection coefficients.

To confirm the proposed design, a 210 *mm* × 210 *mm* sample of the proposed metasurface is fabricated and measured. The experiment setup is depicted in Fig. 9(a), in which the sample is irradiated by one of the horn antennas while the other horn antenna acts as receiver. The receiving antenna can capture both transverse-magnetic (TM) and transverse-electric (TE) modes by placing the antenna on its longer and shorter sides, respectively. Hence both the cross- and co-polarized reflections can be measured. That is to say, when one of the two antennas is perpendicular to each other, the cross-polarized reflection is measured; while the two antennas are parallel to each other, the co-polarized reflection is measured. It should be noted that the center of the two antennas should be placed at the same height. Figure 9(b) gives the measured results, which are limited to 18 GHz due to the restrictions in experimental conditions. Thus only the results from 6 to 18 GHz are presented. Through the comparison of available results, we note that the measurements are consistent with the simulations. The decline during high frequencies is mainly owing to the angle between the antennas. The angle brings about non-normal incidence, which may leads to the worse performance. Meanwhile the inherent vice of the vector network analyzer during high frequencies may also result in such a performance.

**Figure 10:**
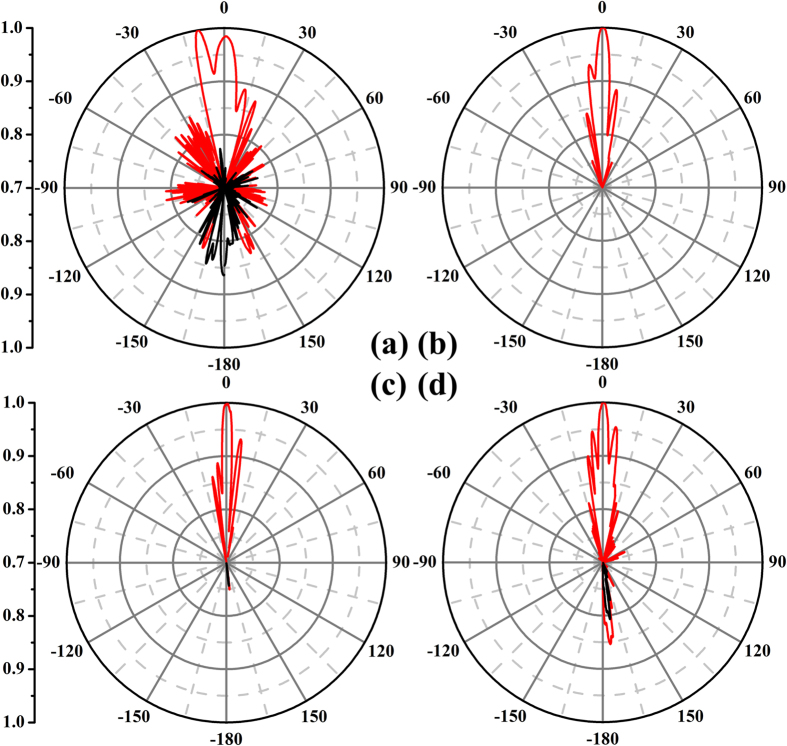
The normalized far-field patterns of the proposed structure, in which the red lines signify the results when using the anchor-shaped structure, while the black lines illustrate the results when using the whole piece of metal as comparison. (**a**) 6 GHz. (**b**) 10 GHz. (**c**) 14 GHz. (**d**) 18 GHz.

To validate the polarization-conversion characteristics, we also measured the radiation patterns of the metasurface, as shoun in Fig. 10, in which the red lines signify the results when using the anchor-shaped structure, while the black lines illustrate the results when using the whole piece of metal as comparison. From Fig. 10, it is obviously that the proposed anchor-shaped ultra-wideband polarization-conversion metasurface performs very well in the working frequency band.

## Discussion

In summary, we have proposed a kind of ultra-wideband polarization-conversion metasurface with the polarization selective and incident angle insensitivity characteristics in the microwave frequency. The bandwidth expansion is realized through multiple resonances. The optimization of the unit cell is fulfilled through the combination of CST Microwave Studio and MATLAB on the basis of genetic optimization algorithm, reducing the complexity in the dimension design of the unit cell. Both the simulated and measured results demonstrate that the proposed metasurface can realize the polarization conversions effectively in ultra wide frequency band. We show that the average polarization conversion efficiency is above 80% in the whole 3 dB bandwidth. When the incident wave is not *x*- or *y*-polarized, the conversion ratio remains low. Such good performance can reduce the interference among the cross-polarized waves in the modern communication system, indicating promising future of the proposed metasurface. The incident angle insensitivity characteristic is another advantage that makes the proposed metasurface user-friendly.

## Methods

Numerical simulations are performed by the combination of commercial software, the CST Microwave Studio and MATLAB. The experimental structure is fabricated on a 3 mm-thick substrate with the relative permittivity 2.65 and loss tangent 0.001, respectively. The thickness of the metal (copper) film is 0.018 *mm*.

**Figure 11:**
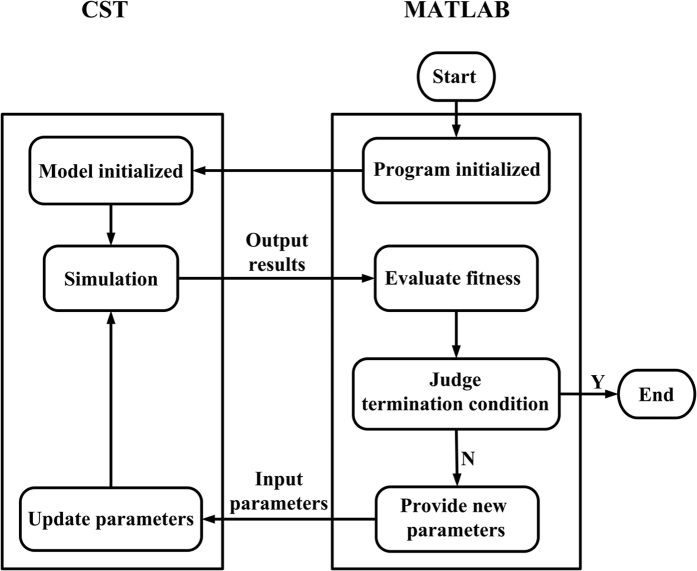


To get the best performance of the metasurface, the genetic optimization algorithm^34^,^35^ is utilized, which is well known and widely used in the optimization techniques. The algorithm is based on the Darwinian theory of natural selection and evolution. The selection, crossover, generation gap, and mutation in the genetic algorithm are applied to a randomly generated initial population iteratively. In the simulations, the mutation probability is set to 0.05, the crossover probability is 0.7, and the generation gap is 0.9, respectively. The optimization flow of the proposed ultra-wideband polarization-conversion metasurface is given by Fig. 11, where Matlab and CST Microwave Studio are combined to find out the optimal design automatically. The period of the unit cell, thickness of the substrate, the operating frequency band, and the permittivity of the dielectric substrate are predefined. The final optimal parameters provided through this procedure are *r* = 3.3 *mm*, *l*_*1*_ = 3.5 *mm*, *w*_*1*_ = 0.5 *mm*, *w*_*2*_ = 0.3 *mm*, *d* = 1*mm*, and *θ* = 140°.

We use the Agilent vector network analyzer and two horn antennas to measure the co- and cross-polarized reflections of the fabricated sample, as illustrated in Fig. 9(a). One of the two horn antennas irradiates the fabricated sample while the other acts as a receiver. The far-field radiation patterns are measured in the microwave chamber as shown in Fig. 12. A copper plate with the same size has been measured for comparison in each experiment.

## Additional Information

**How to cite this article**: Yin, J. Y. *et al.* Ultra Wideband Polarization-Selective Conversions of Electromagnetic Waves by Metasurface under Large-Range Incident Angles. *Sci. Rep.* **5**, 12476; doi: 10.1038/srep12476 (2015).

## Figures and Tables

**Figure 12:**